# Brain Activity in Age-Related Macular Degeneration Patients From the Perspective of Regional Homogeneity: A Resting-State Functional Magnetic Resonance Imaging Study

**DOI:** 10.3389/fnagi.2022.865430

**Published:** 2022-05-09

**Authors:** Qi-Ying Liu, Yi-Cong Pan, Hui-Ye Shu, Li-Juan Zhang, Qiu-Yu Li, Qian-Min Ge, Yi Shao, Qiong Zhou

**Affiliations:** Department of Ophthalmology, Jiangxi Center of National Ocular Disease Clinical Research Center, The First Affiliated Hospital of Nanchang University, Nanchang, China

**Keywords:** neural regional homogeneity, resting state, functional magnetic resonance imaging, age-related macular degeneration (AMD), pathogenesis

## Abstract

**Objective:**

In this study, the regional homogeneity (ReHo) method was used to investigate levels of cerebral homogeneity in individuals with age-related macular degeneration (AMD), with the aim of exploring whether these measures are associated with clinical characteristics.

**Materials and Methods:**

Patients with AMD and healthy controls attending the First Affiliated Hospital of Nanchang University were invited to participate. Resting state functional magnetic resonance images were recorded in each participant and levels of synchronous neural activity were evaluated using ReHo. Receiver operating characteristic (ROC) curves were used to evaluate the sensitivity and specificity of this method.

**Results:**

Eighteen patients with AMD (9 males and 9 females) and 15 healthy controls (HCs) were recruited. The two groups were approximately matched in age, gender and weight. Compared with controls, the ReHo values were significantly higher in the AMD group at the limbic lobe and parahippocampal gyrus, and were significantly reduced at the cingulate gyrus, superior frontal gyrus, middle frontal gyrus, inferior parietal lobule, and precentral gyrus. Mean ReHo values at the cingulate gyrus and the superior frontal gyrus were negatively correlated with clinical symptoms.

**Conclusion:**

Brain neural homogeneity dysfunction is a manifestation of visual pathways in AMD patients, and may be one of the pathological mechanisms of chronic vision loss, anxiety and depression in AMD patients. In addition, the ReHo data may be useful for early screening for AMD.

## Introduction

Age-related macular degeneration (AMD) is a significant cause of irreversible blindness in the elderly (VanNewkirk et al., 2000). AMD affects over one-quarter of those who aged over 75 and is the world’s third most common blinding eye disease, as well as the most common reason for irreversible blindness among the elderly in Western countries (Pennington and DeAngelis, 2016). Asia has recently witnessed an increase in the incidence of AMD and it has been predicted to evolve into a global disease, with the total number of people affected worldwide reaching 196 million by 2020 and increasing to 288 million in 2040 (Wong et al., 2014). The treatment of retinal angiogenesis and fluid leakage in neovascular AMD is currently treated by blocking vascular endothelial growth factor A (Bakri et al., 2019). The mechanisms by which AMD exerts long-term effects on the human brain and behavior are not clear, and there is no cure for this disease. Few researchers have explored the relationship between AMD and spontaneous brain activity. Previous studies using functional magnetic resonance imaging (fMRI) and electroencephalography (EEG) have suggested that synchronous neuronal activity may also make a difference in numerous neurophysiological events (Ward, 2003; Spencer et al., 2004; Li et al., 2015). Resting state fMRI (rs-fMRI) is one of the most effective ways to detect changes in brain activity. Regional homogeneity (ReHo) is a measurement technique used in rs-fMRI to estimate the consistency of signals related to blood oxygen levels between adjacent voxels throughout the brain at rest (Tononi et al., 1998; Zang et al., 2004). ReHo is a method to evaluate brain activity in its resting state, as well as one of the methods currently available to study the partial synchronization of idiopathic fMRI signs. Our previous research using the ReHo method has assessed neurological status in eye diseases including corneal ulcer (Xu et al., 2019), diabetic retinopathy (Liao et al., 2019), optical neuritis (Shao et al., 2015) and others (Dai et al., 2012; Song et al., 2014; Huang et al., 2016a,b, 2017a,2017b; Li et al., 2016, 2020; Tan et al., 2016a,b; Tang et al., 2018; Ye et al., 2018; Zhu et al., 2018; Shao et al., 2019; Xiang et al., 2019; Zhang et al., 2020).

Resting-state fMRI and ReHo values may be useful indicators of macular degeneration at an early stage. In the present study, correlation analysis was used to calculate the average ReHo signal in different brain regions to explore the relationship between the signal and clinical symptoms in AMD patients.

## Materials and Methods

### Subjects

**FIGURE 1:**
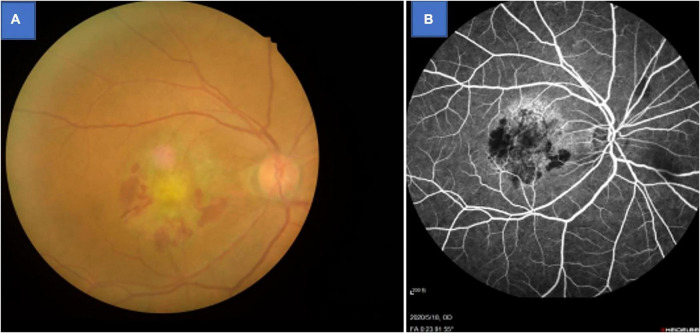
Example of age-related macular degeneration seen on fundus camera **(A)** and fluorescence fundus angiography **(B)**.

**FIGURE 2:**
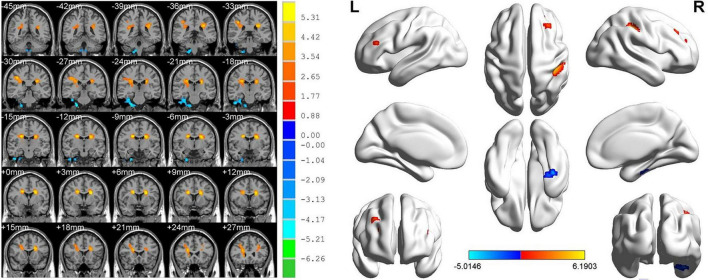
Significant differences in ReHo values between the AMD group and HCs. Blue areas denote significantly reduced ReHo values in the cingulate gyrus, superior frontal gyrus, middle frontal gyrus, inferior parietal lobule, and precentral gyrus, red areas denote significantly increased ReHo values in the limbic lobe and parahippocampal gyrus.

Patients with AMD who regularly visited the ophthalmology department of the First Affiliated Hospital of Nanchang University were invited to participate in this study. Inclusion criteria for AMD patients were: (1) age-related macular degeneration diagnosed using fundus fluorescein angiography and confirmed by indocyanine green angiography (Spectralis HRA-OCT; Heidelberg Engineering, Heidelberg, Germany; Figure 1); (2) no eye disease other than AMD; (3) no anti-vascular endothelial growth factor treatment; (4) no dementia (based on an existing diagnosis, or five or more errors in the Short Portable Mental Status Questionnaire); and (5) no history of brain surgery.

In addition, patients with a history or diagnosis of mild cognitive impairment, Alzheimer’s disease, generalized anxiety disorder, depressive disorder, Parkinson’s disease, proliferative retinopathy, other retinopathy, retinal vein occlusion, neovascular glaucoma, chronic myeloproliferative disease, macular or cystic macular edema were excluded since these conditions may alter the value of the ReHo signals in the brain region associated with macular degeneration.

Healthy controls who met the following criteria were eligible for inclusion: (1) no contraindications to MRI scan (such as implanted metal device); (2) no neurological diseases or psychiatric diseases (such as mania or depression); (3)no prior or present age-related macular degeneration or other retinal or fundus lesions.

The methods used in this study were consistent with the Declaration of Helsinki. The study was approved by the Ethics Committee of the First Affiliated Hospital of Nanchang University. Research protocols and procedures were fully explained to each subject before obtaining written informed consent.

### MRI Parameters

MRI scanning was performed on a 3-Tesla MR scanner (Trio, Siemens, Munich, Germany). T1-weighted images with high resolution were acquired using a tridimensional destruction gradient echo sequence, with repetition time = 190 ms, echo time = 2.26 ms, thickness = 3.0 mm, gap = 0.5 mm, acquisition matrix = 256 × 256, field of view = 250 mm × 250 mm, and flip angle = 9°. Some functional images needed to be corrected at thickness = 4.0 mm, repetition time = 2,000 ms, echo time = 30 ms, gap = 1.2 mm, and field of view = 220 mm × 220 mm, 29 axial.

#### Data Analysis From Functional Magnetic Resonance Imaging

Using MRIcro1 software [MRIcro software (McCausland Center for Brain Imaging, Columbia, SC, United States)],^1^ all images were checked and any defective images removed. The first 10 volumes recorded from each subject were discarded to remove any noise associated with movement at the beginning of the procedure. The valid images were processed using SPM82 and Data Processing Assistant for rs-fMRI DPARSFA (Institute of Psychology, CAS., Beijing, People’s Republic of China) software. Following this, slice timing, head motion correction (any of the six parameters within 1.5 mm or 1.5), and spatial normalization were performed on the digital data. The data were then smoothed using a 6 mm full-width at half-maximum Gaussian. Finally, the fMRI image space was normalized to the Montreal Neurological Institute space employing an echo plane imaging template and was resampled at a resolution of 3 mm × 3 mm × 3 mm. To optimize reliability, the data were de-trended and bandpass filtered (0.01–0.08 Hz) to remove low-frequency drift and physiological high-frequency respiratory and cardiac noise.

### Statistical Analysis

To compare ReHo values between AMD and HC groups, SPM8 software was used to conduct an independent-samples test after excluding other influencing factors such as age and gender (two-tail, voxel level: *P* < 0.005 Gaussian random field correction, cluster-level: *P* < 0.05, cluster: 162).

#### Brain–Behavior Correlation Analysis

Regions of interest were defined on images from each group using REST software.^2^ Within each region, the average ReHo value was obtained from the ReHo values of all voxels. Correlation analysis were used to determine whether the ReHo values were associated with clinical manifestations (*P* < 0.05 was considered statistically significant).

### Clinical Data Analysis

Intraocular pressure, best-corrected visual acuity and body weight were measured in each participant, and these plus disease duration were recorded. Demographic and clinical variables were compared between the two groups using SPSS version 20.0 software, and *P*-values < 0.05 were again considered significant. Receiver operating characteristic (ROC) curves were used to test stability, sensitivity and specificity.

## Results

### Demographics and Behavioral Results

The two groups were statistically similar in weight (*P* = 0.542) and age (*P* = 0.785), but significantly poorer monocular visual acuities were found in the AMD than HC group (right *P* = 0.003; left *P* = 0.004) (Table 1).

**TABLE 1:** Demographics and clinical measurements of AMD and HC Groups.

Condition	AMD	HC	*t*	*P*-value*
Male/female	10/8	10/8	N/A	>0.99
Age (years)	55.25 ± 4.04	53.87 ± 5.16	0.375	0.785
Weight (kg)	61.58 ± 11.84	69.36 ± 12.78	0.542	0.542
Handedness	18R	18R	N/A	>0.99
Best-corrected VA-L	0.15 ± 0.10	1.05 ± 0.10	−4.836	0.004
Best-corrected VA-R	0.10 ± 0.05	1.05 ± 0.15	−4.736	0.003
Duration of AMD (months)	3.34 ± 2.88	N/A	N/A	N/A
IOP-L	12.14 ± 3.64	14.36 ± 3.76	0.312	0.898
IOP-R	14.26 ± 3.97	15.95 ± 4.12	0.336	0.802

Independent t-tests comparing the two groups (p < 0.05 represented statistically significant differences). Data shown as mean standard deviation or n. AMD, age-related macular degeneration; HC, healthy control; L, left; R, right; N/A, not applicable; VA, visual acuity; IOP, intraocular pressure. *p < 0.05 represented statistically significant differences.

### Regional Homogeneity Value Comparisons Between Groups

**FIGURE 3:**
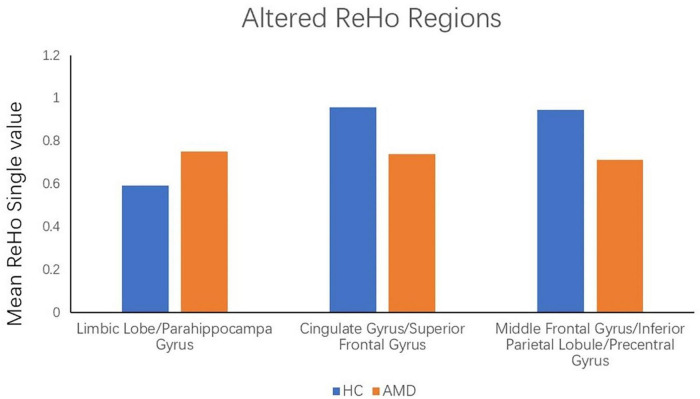
The mean single ReHo value between the AMDs group and HCs. Data presented as mean ± standard deviation. ReHo, regional homogeneity; HCs, healthy controls; N/A, not applicable; AMD, age-related macular degeneration.

ReHo values in the AMD group were significantly higher than controls at the limbic lobe and parahippocampal gyrus (*P* < 0.05), and significantly lower at the cingulate gyrus, superior frontal gyrus, middle frontal gyrus, inferior parietal lobule and precentral gyrus (*P* < 0.05) (Figures 2, 3 and Table 2).

**TABLE 2:** Brain regions with significantly different ReHo values between the AMDs and HCs.

Brain areas	MNI coordinates			Number of voxels	*T*-value	ROI 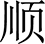 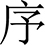 ()
	X	Y	Z			
**HC < AMD**						
Limbic Lobe/Parahippocampal Gyrus	27	−27	−48	541	−5.0146	1
**HC > AMD**						
Cingulate Gyrus/Superior Frontal Gyrus	21	6	27	832	6.1903	2
Middle Frontal Gyrus/Inferior Parietal Lobule/Precentral Gyrus	−21	6	24	661	5.9303	3

*Voxel-level: P < 0.005, GRF correction, cluster-level: 162. P < 0.05. CU, corneal ulcer; HCs, healthy controls; BA, Brodmann area.*

### Receiver Operating Characteristic Curve

**FIGURE 4:**
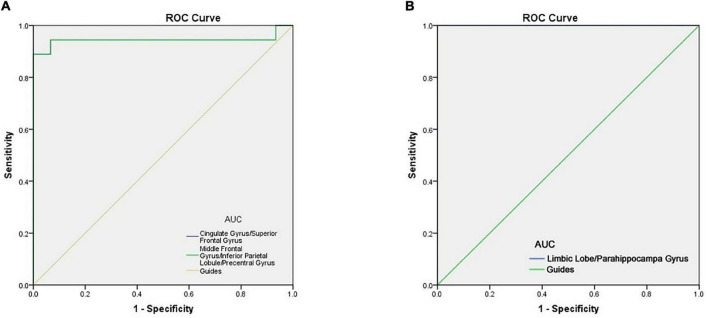
ROC curve analysis of the mean ReHo values for altered brain regions. **(A)** The area under the ROC curve were 0.944 (*p* < 0.0001; 95% CI: 0.845–1.000), for Cingulate Gyrus/Superior Frontal Gyrus, Middle Frontal Gyrus/Inferior Parietal Lobule/Precentral Gyrus 0.944 (*p* < 0.0001; 95% CI: 0.845–1.000). **(B)** The area under the ROC curve were 1.000 (*p* < 0.0001; 95% CI: 1.000–1.000), for Limbic Lobe/Parahippocampal Gyrus. AUC, area under the curve; ROC, receiver operating characteristic.

Since ReHo values differed between groups, as explained above, they were further analyzed using ROC curves to assess how well these values distinguish between the two groups. AUC (Area Under Curve) is defined as the area under the ROC curve enclosed by the coordinate axis, The closer the AUC is to 1.0, the higher the authenticity of the detection method AUCfor ReHo values at the cingulate gyrus, superior frontal gyrus, middle frontal gyrus, inferior parietal lobule and precentral gyrus was 0.944 in each case (AMDs > HCs) (Figure 4B). ROC curve can also reflect to some extent that ReHo values have certain advantages in diagnosing AMD (Figure 4A).

### Correlation Analysis

**FIGURE 5:**
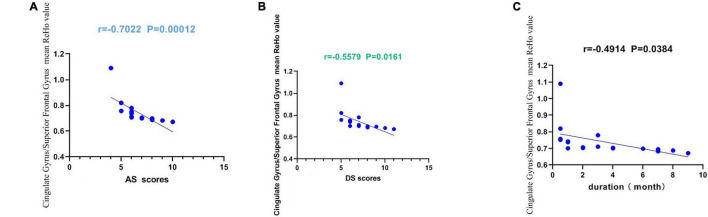
Correlations between the mean ReHo values of the Cingulate Gyrus/Superior Frontal Gyrus and the clinical behaviors. **(A)** The AS showed a negative correlation with the mean ReHo values of the Cingulate Gyrus/Superior Frontal Gyrus (*r* = −0.702, *p* = 0.0001 < 0.001), **(B)** the DS showed a negative correlation with the mean ReHo values of the Cingulate Gyrus/Superior Frontal Gyrus (*r* = −0.5579, *p* = 0.0161 < 0.05). **(C)** The duration showed a negative correlation with the mean ReHo values of the Cingulate Gyrus/Superior Frontal Gyrus (*r* = 0.4914, *p* = 0.0384 < 0.05). AS, anxiety scores; DS, depression scores.

The present study used the Chinese version of the Hospital Anxiety and Depression Scale (HADS). The HADS questionnaire, 10 which involves self-assessment, has been found to be a reliable instrument for determining depression and anxiety status in a hospital outpatient clinic setting. The anxiety and depressive subscales are also valid measures of the severity of emotional disorder. We defined that a score greater than or equal to 8 points was positive. The higher the score, the more serious the depression and anxiety. Within the AMD group, anxiety scores, depression scores and disease duration were all inversely correlated with the ReHo values at the cingulate gyrus and at the superior frontal gyrus (*P* < 0.05). These data indicate that AMD is associated with all three factors (Figure 5).

## Discussion

Our previous studies on the ReHo method have demonstrated that it can be applied to a variety of ophthalmic diseases and has broad scope for further development (Table 3). The studies have highlighted regional disease-related changes in brain activity and their potential effects that needed to be further examined (Table 4). So far, there is no consensus on the relationship between ReHo value and resting state of AMD patients, and the present study aimed to fill this gap.

**TABLE 3:** Regional homogeneity method applied in ophthalmological diseases.

References	Year	Disease
Xu et al. (2019)	2019	Corneal ulcer
Liao et al. (2019)	2019	Diabetic retinopathy
Huang et al. (2017a)	2017	Late monocular blindness
Xiang et al. (2019)	2019	Classic trigeminal neuralgia
Tang et al. (2018)	2018	Acute eye pain
Huang et al. (2017b)	2017	Retinal detachment
Huang et al. (2016a)	2016	Universal acute open-globe injury
Zhang et al. (2020)	2020	Diabetic vital pathogenesis
Shao et al. (2015)	2015	Optical neuritis
Huang et al. (2016b)	2016	Comitant strabismus
Shao et al. (2019)	2019	Strabismus and amblyopia
Song et al. (2014)	2014	Glaucoma
Dai et al. (2012)	2012	Sleep disorders
Li et al. (2016)	2016	Parkinson’s disease

**TABLE 4:** Brain regions alternation and its potential impact.

Brain regions	Experimental result	Brain function	Anticipated results
Limbic Lobe	HC < AMD	Processing of memory, decision-making and emotional responses	Depression, epilepsy, affective cognitive impairment
Parahippocampal Gyrus	HC < AMD	Associative memory, source memory and processing of emotional stimuli	The problems of memory, sleep
Cingulate Gyrus	HC > AMD	The integration of attention and emotional information	Disorders of emotion regulation
Superior Frontal Gyrus	HC > AMD	Part of the default model network	Depression and anxiety
Middle Frontal Gyrus	HC > AMD	Part of the default model network	Depression and anxiety
Inferior Parietal Lobule	HC > AMD	Part of the default model network	Depression and anxiety
Precentral Gyrus	HC > AMD	Control voluntary movement	Depressive disorder and memory performance

**FIGURE 6:**
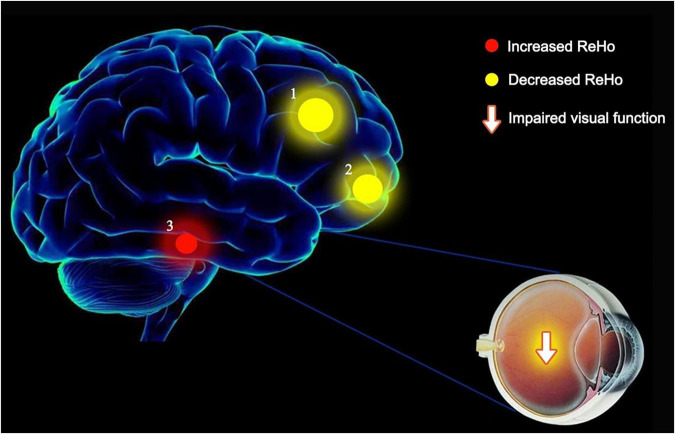
The ReHo results of brain activity in the AMD group. Compared with the HCs, the ReHo values of the following regions were decreased to various extents: 1- Cingulate Gyrus/Superior Frontal Gyrus (BA 32, *t* = 6.1903), 2- Middle Frontal Gyrus/Inferior Parietal Lobule/Precentral Gyrus (*t* = 5.9303). Compared with the HCs, the ReHo values of the following regions were increased to various extents: 3- Limbic Lobe/Parahippocampal Gyrus (*t* = −5.0146). HCs, healthy controls; BA, Brodmann’s area.

We found that ReHo values were significantly different between AMD patients and controls, being higher in AMD at the limbic lobe and parahippocampal gyrus and lower at the cingulate gyrus, superior frontal gyrus, middle frontal gyrus, inferior parietal lobule and the precentral gyrus (Figure 6).

### Implications of Increased Regional Homogeneity Values in Age-Related Macular Degeneration

**FIGURE 7:**
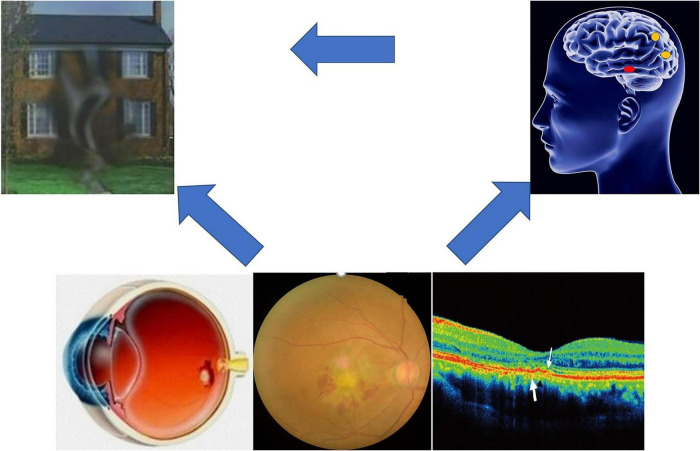
Correlations between AMDs ReHo and behavioral performance, compare with HCs, AMDs may suffer more problems with dealing with emotion, memory and visual disturbances.

The limbic system plays a major part in memory, decision making and emotional feedback. Research has shown that its damage interferes with memories that are enhanced by emotion (Amunts et al., 2005) and that the limbic system is associated with depression and anxiety in the epilepsies (Krishnan, 2020). The brain combines emotion with cognition to produce flexible behavioral output based on its judgments of the environment (Rusbridge, 2020). In addition, the parahippocampal gyrus is associated with episodic memory relating to source memory, associative memory and processing of emotional stimuli (Suthana et al., 2012). For example, Mankin found that deep brain stimulation of hippocampal circuits can modulate human memory (Mankin and Fried, 2020). In addition to its role in memory, the parahippocampal cortex is involved in visuospatial processing related to scene perception and spatial representation of navigation (Aminoff et al., 2013). However, further study is needed to confirm whether an increase in the value of ReHo in the parahippocampal gyrus in AMD has an effect on memory enhancement (Figure 7).

### Implications of Decreased Regional Homogeneity Values in Age-Related Macular Degeneration

Anatomically, the anterior central gyrus, also called precentral gyrus, is divided into four parts by three contours in the paracentral lobule and gyrus. It is bounded above by the anterior central sulcus and below by the lateral fissure, which is mainly located on the lateral side of the cerebral hemisphere. Study has shown that abnormal or weak connections between them may be risk factors for disease (Nebel et al., 2014). According to previous studies, the paracentral gyrus has also been associated with memory ability and depression disorders (Nebel et al., 2014; Li et al., 2018; Shang et al., 2018).

The structure on the medial side of the cerebral hemisphere between the cingulate sulcus and the corpus callosum sulcus is called the cingulate gyrus. It belongs to the cortical part of the limbic system and is an important region connecting the orbitofrontal cortex, amygdala, insular lobe, septal nucleus, and hypothalamus. The cingulate gyrus is the bridge between attention and emotional processing and is responsible for the integration of attention and emotional information. Burger’s research points out that cingulate gyrus activation was sharply reduced in major depressive disorders (Nebel et al., 2014) potentially indicating impaired bottom-up emotional processing and abnormal automatic emotional regulation. The Fischer’s study suggested that parietal activity may be particularly important for linking long-term memory representation and attention components (Bürger et al., 2017). In other fMRI study, frontal and parietal activation was found in spatial memory-guided attention tasks (Fischer et al., 2021).

To some extent, the decline of memory and depression in AMD patients can be traced to the changes in brain activity (Giesbrecht et al., 2013). The present results showed that the ReHo value of five brain regions were decreased in AMD, with reliability verified by the ROC curve analysis results. AUC values of over 0.7 are considered high, and our analysis showed that the AUC values of ReHo values in the above brain regions were all greater than 0.9, indicating very high accuracy. The abnormality of ReHo values in some brain regions is an important finding relating to the diagnosis of AMD based on imaging data. The results suggest that the ReHo method may be a non-invasive, rapid and sensitive method for early diagnosis of AMD patients in the future.

## Conclusion

The present findings suggest that AMD patients have abnormal spontaneous brain activity, which may prove useful for early disease detection. Activity in the cingulate gyrus and superior frontal gyrus was inversely associated with anxiety, depression and disease duration. These findings provide powerful information for further research. However, there are still some limitations in our study. Such as larger sample sizes and detailed grouping of different types of AMDs are needed. Moreover, our study only demonstrated the existence of the correlation between changes of ReHo values in specific brain regions and RVO. But It is unclear whether AMD will cause changes in brain activity or whether patients with brain dysfunction are susceptible to AMD.

## Data Availability Statement

The original contributions presented in the study are included in the article/supplementary material, further inquiries can be directed to the corresponding author/s.

## Ethics Statement

The studies involving human participants were reviewed and approved by the First Affiliated Hospital of Nanchang University. The patients/participants provided their written informed consent to participate in this study.

## Author Contributions

Q-YiL, Y-CP, H-YS, YS, and QZ: study design and manuscript preparation. Q-YiL, Y-CP, H-YS, L-JZ, YS, and QZ: data collection. Q-YiL, Y-CP, H-YS, Q-YuL, YS, and QZ: statistical analysis. Q-YiL, Y-CP, H-YS, Q-MG, YS, and QZ: data interpretation and literature search. All authors read and approved the final manuscript.

## Conflict of Interest

The authors declare that the research was conducted in the absence of any commercial or financial relationships that could be construed as a potential conflict of interest.

## Publisher’s Note

All claims expressed in this article are solely those of the authors and do not necessarily represent those of their affiliated organizations, or those of the publisher, the editors and the reviewers. Any product that may be evaluated in this article, or claim that may be made by its manufacturer, is not guaranteed or endorsed by the publisher.

## Footnotes

1
www.mricro.com

2
http://www.restfmri.net
